# Growth Control of Adherent-Invasive *Escherichia coli* (AIEC) by the Predator Bacteria *Bdellovibrio bacteriovorus*: A New Therapeutic Approach for Crohn’s Disease Patients

**DOI:** 10.3390/microorganisms8010017

**Published:** 2019-12-20

**Authors:** Giulia Bonfiglio, Bruna Neroni, Giulia Radocchia, Arianna Pompilio, Francesco Mura, Maria Trancassini, Giovanni Di Bonaventura, Fabrizio Pantanella, Serena Schippa

**Affiliations:** 1 Department of Public Health and Infectious Diseases, Microbiology section, Sapienza University of Rome, 00185 Roma, Italy; giulia.bonfiglio@uniroma1.it (G.B.); bruna.neroni@uniroma1.it (B.N.); giulia.radocchia@uniroma1.it (G.R.); maria.trancassini@uniroma1.it (M.T.); fabrizio.pantanella@uniroma1.it (F.P.); 2 Department of Medical, Oral and Biotechnological Sciences, and Center for Advanced Studies and Technology (CAST), University “G. d’Annunzio” of Chieti-Pescara, 66100 Chieti, Italy; arianna.pompilio@unich.it (A.P.); gdibonaventura@unich.it (G.D.B.); 3 Electrical and Energy Engineering, Sapienza Nanoscience & Nanotechnology Laboratories (SNN-Lab), ‘Sapienza’ University of Rome, 00185 Roma, Italy; francesco.mura@uniroma1.it

**Keywords:** microbiota, predation, Crohn’s disease, *Escherichia coli*, *Bdellovibrio bacteriovorus*

## Abstract

In Crohn’s disease (CD) patients, intestinal dysbiosis with an overgrowth of Proteobacteria, mainly *Escherichia coli*, has been reported. A new pathotype of *E. coli*, the adherent-invasive *Escherichia coli* strain (AIEC), has been isolated from the mucosae of CD patients. AIEC strains play an important role in CD pathogenesis, increasing intestinal mucosa damage and inflammation. Several studies have been undertaken to find possible strategies/treatments aimed at AIEC strain reduction/elimination from CD patients’ intestinal mucosae. To date, a truly effective strategy against AIEC overgrowth is not yet available, and as such, further investigations are warranted. *Bdellovibrio bacteriovorus* is a predator bacterium which lives by invading Gram-negative bacteria, and is usually present both in natural and human ecosystems. The aim of this study was to evaluate a novel possible strategy to treat CD patients’ mucosae when colonized by AIEC strains, based on the utilization of the Gram-negative predatory bacteria, *B. bacteriovorus*. The overall results indicate that *B. bacteriovorus* is able to interfere with important steps in the dynamics of pathogenicity of AIEC strains by its predatory activity. We indicate, for the first time, the possibility of counteracting AIEC strain overgrowth by exploiting what naturally occurs in microbial ecosystems (i.e., predation).

## 1. Introduction

Inflammatory bowel disease (IBD) includes a group of gastrointestinal tract diseases, of which the main types are Crohn’s disease (CD) and ulcerative colitis (UC). The IBD etiology has not yet been completely explained; however, it is now widely recognized that the intestinal microbiota is an effective factor, playing an important function in the triggering and development of CD [1]. CD patients have been shown to demonstrate a decrease of intestinal microbial biodiversity and a concomitant increase of the phylum Proteobacteria, mainly the Enterobacteriaceae family—particularly *Escherichia coli* species [2]. A new pathotype of *E. coli*, the adherent-invasive *E. coli* (AIEC), whose prototype is the strain LF82, has been isolated for the first time in active CD patients’ intestinal mucosae [3,4,5,6,7,8,9]. AIEC strains, a favorite in the CD intestinal habitat, are able to adhere and invade the intestinal epithelial cells [10], and can persist and reproduce inside macrophages without stimulating apoptosis, but inducing tumour necrosis factor alpha release [11]. The presence of AIEC strains in subjects not affected by IBD has indicated the “pathobiont” nature of these strains [12]. The growth level of AIEC strains colonizing healthy subjects is kept under control by the intestinal microbial ecosystem. Moreover, they are not able to translocate through the mucosal barrier, due to its perfect integrity [13]. When inflamed intestinal conditions take over, as is the case in CD patients, such genetic variants take advantage and become overgrown [12,14]. The presence of AIEC strains in CD patients’ mucosae can increase inflammation and enhance mucosal damage [11,15,16]. Studies aimed at improving IBD patients’ quality of life have been conducted with the purpose of devising new strategies to reduce or control the growth of AIEC strains. The pathogenicity mechanism of AIEC is linked to its adhesion and invasion ability, and to its ability to overgrow, taking advantage of inflamed mucosae where upregulation of CEACAM6 (carcinoembryonic antigen-related cell-adhesion molecule) and of the chaperone Gp96 (the endoplasmic reticulum-stress response) occurs [17]. To date, all of the proposed strategies have aimed to block those steps [18,19,20], but an effective approach to counteract AIEC overgrowth is not yet available, and additional research is needed. In the present work, we explore the possibility of neutralizing the overgrowth of AIEC strains by exploiting the predation phenomena that naturally occur in microbial ecosystems. Bacteria in their habitat are preyed upon by bacteriophages and prokaryotic predators [21]. *B. bacteriovorus* is a small (0.2–0.5 μm × 0.5–2.5 μm) aerobic or facultative anaerobic Gram-negative bacterium belonging to the class of Delta-Proteobacteria, and is a predator of other Gram-negative bacteria. *B. bacteriovorus* can be found in several habitats (terrestrial, marine, and biotic) where it plays the role of “ecological balancer” that, through its predatory activity, preserves stability in the ecosystem. It has also recently been demonstrated to colonize the intestinal mucosae of healthy human subjects [22,23]. *B. bacteriovorus* has the natural ability to predate Gram-negative bacteria; it swims using its long flagellum, collides with its prey, and invades its periplasmic space, where it undergoes a complex replication cycle culminating in killing the prey and releasing its progeny [24,25]. *B. bacteriovorus* is considered by many authors to be an *amphibiotic* microorganism which is able to perform a dual activity—that is, probiotic activity, ensuring a balance in the ecosystem in which it is present, and antibiotic activity, attacking and killing pathogenic Gram-negative bacteria species, both in planktonic and biofilm form [26].

The present study aimed to evaluate the use of the Gram-negative predatory bacteria *B. bacteriovorus* to control or eliminate adhesive AIEC strains by interfering with crucial aspects of their pathogenicity. Toward this aim, the predatory activity of *B. bacteriovorus* was first applied to AIEC strains, in both planktonic and biofilm forms. Subsequently, we assessed whether *B. bacteriovorus* could interfere with the adhesive/invasive capabilities of AIEC strains in an intestinal Caco-2 cell line. Further, the greater wax moth *Galleria mellonella*, recently introduced as an alternative and highly-predictive model with which to study bacterial diseases, allowed us to evaluate the in vivo predatory capabilities of *B. bacteriovorus* on AIEC. Finally, we documented the predatory activity of *B. bacteriovorus* against AIEC using field emission scanning electron microscopy.

## 2. Materials and Methods 

### 2.1. Bdellovibrio bacteriovorus Growth Conditions

The *Bdellovibrio bacteriovorus* strain HD100 (DSM No.:50701), acquired from the Leibniz Institute DSMZ-German Collection of Microorganisms and Cell Cultures GmbH (Braunschweig, Germany), was conserved at −80 °C in glycerol stocks. The predatory bacterium was cultured and processed as previously described [27]. Briefly, 50 μL of glycerol stock was seeded onto a double-layered plate of YPSC medium (0.25 g/L Mg_2_SO_4_, 0.5 g/L of sodium acetate, 1 g/L broad bean peptone, 1 g/L yeast extract, 0.25 g/L of CaCl_2_ × 2H_2_O) where 6 g/L of agar and 1 × 10^8^ CFU/mL of prey cells (*E. coli* LF82) was added to the top layer, and 10 g/L of agar was added to the bottom layer. The double-layer plates were incubated at 30 °C for 3–4 days (until *B. bacteriovorus* growth was visible as clear plaque). 

### 2.2. Adherent-Invasive *Escherichia coli* (AIEC) Growth Conditions

AIEC strain LF82, already present in our collection, was conserved at −80 °C in glycerol stocks. It was directly seeded from glycerol stock LF82 on a Brain Heart Infusion (BHI) agar (Thermo Scientific Oxoid microbiology product, Basingstoke Hampshire, UK) plate and grown overnight at 37 °C. One-to-three colonies of LF82 grown on the BHI agar plate were used to inoculate 10 mL of BHI broth (Thermo Scientific), and incubated overnight at 37 °C with agitation (180 rpm). The growth was spectrophotometrically evaluated (BioPhotometer, Eppendorf, Hamburg, Germany). 

### 2.3. Predator Stock Lysates Preparation

Predator stock lysates were made by coculturing the predator and the prey (*E. coli* LF82, 10^8^ CFU/mL) inoculating pieces of YPSC medium double-layered plate in Diluted Nutrient Broth 2× (DNB2×) (Bacto Nutrient Broth 1.6 g/L, yeast extract 0.1 g/L, casaminoacids 0.5 g/L, CaCl_2_ × 2H_2_O 0.3 g/L, MgCl_2_ × 6H_2_O 0.6 g/L) [28]. The coculture was then incubated at 30 °C on a rotary shaker for at least 72 h, until the cultures cleared. The fresh co-culture was filtered three times with 0.45-μm pore-size filters (Millex^®^, Merck KGaA, Darmstadt, Germany) to eliminate the prey cells. In order to ensure the effective elimination of the LF82, aliquots (10 µL) of the filtered co-culture were plated onto BHI agar plates. Further, the filtrated co-culture was washed three times at 29,000× *g* for 45 min (Sorvall LYNX 4000 centrifuge, Thermo Fisher Scientific Inc), re-suspending the pellet in 10 mL of phosphate-buffered saline (PBS) (Sigma-Aldrich, Merck KGaA, Darmstadt, Germany) for two cycles, and then in 2 mL of PBS after the last step. The predator concentration was then evaluated by counting the plaque-forming units (PFU) and seeding the *B. bacteriovorus* preparation onto a double-layered plate of YPSC medium as described above. We obtained a PFU count of between 5 × 10^8^ and 5 × 10^9^ PFU/mL. The predator stock was prepared fresh for each experiment.

### 2.4. Predation Assays on Planktonic Cultures

Predation assays on planktonic cultures were carried out as previously described [29], with minor modifications. Briefly, 20 mL of Ca/HEPES (5.9 g/L HEPES free acid; 0.28 g/L CaCl_2_ × 2H_2_O) containing 1 × 10^8^ CFU/mL of LF82 and 2 mL of fresh predator *B. bacteriovorus* stock lysates suspension (1 × 10^8^ PFU/mL) in PBS were incubated for 48 h at 37 °C. A control culture of LF82 at 1 × 10^8^ CFU/mL, added with 2 mL of PBS, was prepared. Two control cultures of a nonpathogenic *E. coli* strain MG1655 (1 × 10^8^ CFU/mL) in 20 mL of Ca/HEPES, treated and not treated with 2 mL of fresh predator *B. bacteriovorus* stock lysates suspension (1 × 10^8^ PFU/mL) in PBS, were also evaluated. Aliquots (500 µL) of the cultures were collected at time 0, 30 min, 3 h, 6 h, 24 h, and 48 h. One hundred microliters were diluted and used for counting the viable cells of LF82 on the TSA plates (Thermo Scientific Oxoid, UK), and the rest (400 µL) was filtered three times with a 0.45-μm pore-size filter and plated onto a double-layered YPSC medium plate to determine the predator concentration. Predatory assays were performed in triplicate. The statistical significance of the results was assessed using GraphPad Prism software (version 5.03 for Windows, San Diego California USA, www.graphpad.com) (two-way ANOVA, Holm–Sidak’s multiple comparison test), considering *p*-values < 0.05 as statistically significant.

### 2.5. Evaluation of *B. bacteriovorus* Impact on Preformed LF82 Biofilm

Two hundred microliters of an overnight LF82 culture in BHI broth at OD_600_ = 1 were used to inoculate two 96-well microtiter plates. The plates were incubated at 37 °C for 72 h. After the washing step with PBS, 200 µL per well of freshly-prepared *B. bacteriovorus* stock lysate was added to one plate, and a new incubation step of 24 h at 37 °C was carried out. The second 96-well plate was left as predator-free control, to which 200 µL of PBS were added, followed by incubation for 24 h at 37 °C, to evaluate the amount of biofilm production without interferences. At the same time, the nonpathogenic *E. coli* MG1655 culture in BHI broth at OD_600_ = 1 was used to inoculate the other two 96-well microtiter plates, which were treated as described for LF82 strain. 

After incubation, both plates were washed with PBS, and then crystal violet staining was performed [28]. All experiments were conducted three times independently. LF82 was considered positive for biofilm formation at OD_570_ values ≥ 0.12. The statistical significance of the results was evaluated using GraphPad Prism software (nonparametric test, Mann–Whitney test), considering *p*-values < 0.05 as statistically significant.

### 2.6. Evaluation of *B. bacteriovorus* Impact on LF82 Biofilm Formation 

Two 96-well plates were inoculated with 200 µL of a coculture of prey (10^8^ CFU/mL) and predator (10^8^ PFU/mL) in BHI broth. For a control of LF82 biofilm production, as predator-free control, two 96-wells plates were inoculated with an LF82 culture (10^8^ CFU/mL) and added with 200 µL of PBS. At the same time, two 96-well plates were inoculated with 200 µL of a co-culture of MG1655 (10^8^ CFU/mL) as prey, and predator (10^8^ PFU/mL) in BHI broth and treated as described for LF82 strain. The plates were incubated at 37 °C, and the biofilm production was evaluated as described above, at 48 and 72 h. All experiments were conducted three times independently. The statistical significance of the results was evaluated using GraphPad Prism software (nonparametric test, Mann–Whitney test), considering *p*-values < 0.05 as statistically significant.

### 2.7. Caco-2 Cell Line Cultivation 

The human colorectal adenocarcinoma Caco-2 epithelial cell line was obtained from the American Type Culture Collection (ATCC^®^ HTB-37 ™) and stored in liquid nitrogen. Caco-2 cells were cultured in Dulbecco’s Modified Eagle Medium (DMEM, Corning^®^, New York, USA) supplemented with 10% fetal bovine serum (FBS), 1% nonessential amino acids, and antibiotics to a final concentration of 100 U/mL penicillin and 100 μg/mL streptomycin (Corning^®^, New York, NY, USA). Cultures were incubated at 37 °C in a humidified 5% CO_2_ atmosphere. In the adhesion and invasion assays, cells were seeded in 24-well culture plates at a concentration of 2 × 10^5^ cells/mL. Experiments were performed three days post seeding, to reach a Caco-2 monolayer. The culture medium was replaced after two days and medium without antibiotics was used for the last medium change. 

### 2.8. Bacterial Adhesion Assay

The suspension of the predator (50 μL per well, 10^8^ PFU/mL in PBS), prepared as previously described, was used to treat Caco-2 cells for 2 h at 37 °C. After incubation, the cells were washed twice with sterile PBS and then infected with LF82 at a concentration of 2 × 10^7^ CFU/mL, corresponding to a multiplicity of infection (MOI) of 100:1 for Caco-2 cells, in DMEM medium without antibiotics, at 37 °C. As positive control, the cells were infected with only LF82 (2 × 10^7^ CFU/mL), without pretreatment with the predator. At the same time, to have a negative control of adhesion and invasion assay, untreated Caco-2 cells were infected with the nonpathogenic MG1655 at a concentration of 2 × 10^7^ CFU/mL, corresponding to a multiplicity of infection of 100:1 [12]. After incubation, non-adhered bacteria were removed by washing the cell cultures twice with PBS. Cells with adhered bacteria were detached with 250 μL of trypsin-EDTA (Corning^®^) per well for 5 min at 37 °C, followed by addition of 750 μL culture medium containing FBS to stop the trypsin reaction [30]. Serial 10-fold dilutions were prepared in PBS and plated onto brain heart infusion (BHI) agar (Thermo Scientific) at 37 °C for 24 h to obtain the vital bacterial count. The colonies were counted and compared to the number of initial bacteria inoculated in the well and expressed as a percentage for statistical analysis. All statistical analyses were performed using GraphPad Prism statistical software (Mann–Whitney test), considering *p*-values < 0.05 as statistically significant.

### 2.9. Bacterial Invasion Assay

The ability of *B. bacteriovorus* to interfere with LF82 capacity to invade Caco-2 cells was determined using the gentamicin-protective assay [30]. Briefly, the Caco-2 cell layers—treated and untreated (as previously described in the adhesion assay)—were infected for two hours with LF82 at an MOI of 100:1. At the same time, untreated Caco-2 cells were infected with the non-pathogenic MG1655 at a concentration of 2 × 10^7^ CFU/mL, corresponding to an MOI of 100:1, in order to have an invasion negative control. Then, infected cells were washed twice with PBS before the addition of 500 μL/well DMEM containing 150 μg/mL gentamicin (Sigma-Aldrich Chemie GmbH, Buchs, Switzerland) and incubated at 37 °C for an additional hour in order to kill extracellular bacteria. After two washing steps with PBS, 250 μL of trypsin-EDTA was added, followed by another incubation step for 5 min at 37 °C. Intestinal cells were then lysed by adding 250 μL 0.1% (*v*/*v*) Triton X-100 (Sigma) per well and incubated a 37 °C for 10 min [30]. After incubation, samples were collected, adding 500 μL culture medium containing FBS, and seeded on BHI agar to determine the invasive LF82 count, as described above for the adhesion. The adhesion-invasion experiments were performed simultaneously and repeated three times. All statistical analyses were performed using GraphPad Prism statistical software (Mann–Whitney test), considering *p*-values < 0.05 as statistically significant. To test the possible cytotoxicity of *B. bacteriovorus* on Caco-2 cell monolayer, a trypan blue dye exclusion assay of cells pretreated with *B. bacteriovorus*, and control cells that were not pretreated, was performed. Cells were counted and the viable cells percentage was calculated [31,32].

### 2.10. In Vivo Evaluation of *B. bacteriovorus* Toxicity 

The mortality caused by *B. bacteriovorus* was assessed in vivo using *G. mellonella* wax-moth larvae. For each group, 20 larvae weighing 250–350 mg were injected using a Hamilton syringe (Sigma-Aldrich) directly into the hemocoel via the left proleg with 10 µL of a standardized inoculum (corresponding to 1.0 × 10^5^ PFU/larva) prepared in PBS. Control larvae were inoculated with vehicle (PBS) only. Larvae were incubated into a Petri dish at 37 °C, and the number of dead caterpillars was counted daily until 96 h, considering as dead those unresponsive to touch and to gentle shaking of the Petri dish.

### 2.11. In Vivo Protection Studies against *E. coli* Infection 

First, a dose–response curve was generated to determine the optimum inoculum for larval killing (i.e., LD_50_ at 24 h post inoculation). *G. mellonella* larvae (*n* = 20/group) were infected with *E. coli* LF82 at several infectious doses (10^4^, 10^5^, 10^6^, 10^7^, 5 × 10^7^, 10^8^, and 5 × 10^8^ CFU/larva) via a 10-µL injection into the left proleg. Larvae were then incubated at 37 °C and scored for survival daily until 96 h. Control larvae were administrated with vehicle (PBS) only. Next, in the protection studies, larvae (*n* = 20/group) were first administered with *B. bacteriovorus* at 1.0 × 10^5^ PFU/larva, and after 30 min they were infected with *E. coli* LF82 at LD_50_ (5.0 × 10^7^ CFU/larva), corresponding to an MOI of 1:500 (prey:predator), by a second injection into the hemocoel via the right proleg. Positive control larvae were first administered with 10 µL PBS into the left proleg, and after 30 min were infected with *E. coli* LF82. Negative control larvae, aimed at evaluating the injury caused by the double injection, were administered twice with 10 µL PBS, 30 min apart. Larvae were incubated at 37 °C and scored for survival daily until 96 h. Data from duplicate experiments were pooled to give *n* = 40. These pooled survival data were plotted using the Kaplan–Meier method and differences in survival were calculated using the log-rank (Mantel–Cox) test, with a *p*-value < 0.05 indicating statistical significance. All statistical analyses were performed using GraphPad Prism, version 7.0 (GraphPad Software Inc., San Diego, CA, USA).

### 2.12. Field Emission Scanning Electron Microscopy (FESEM) 

Preformed 72-h-old LF82 biofilm was allowed to form on two silicon slides. One was added with *B. bacteriovorus* suspension (10^8^ PFU/mL) in PBS and incubated for 24 h at 37 °C, and the other was treated with PBS. The silicon slides were subsequently rinsed with sterile saline solution (SS) and fixed with glutaraldehyde in SS (2% (*v*/*v*)) at 25 °C for 5 h in the dark. After this step, silicon slides were washed first in SS, and subsequently in distilled water. A post-fixing step in 1% osmium tetroxide aqueous solution for 24 h at 4 °C in the dark was then carried out. Samples were sequentially dehydrated using increasing concentrations of ethanol/water (30%, 50%, 70%, 90%, 99% (*v*/*v*)) for 15 min, dried and observed by FESEM using the Zeiss Auriga 405 apparatus (Carl Zeiss AG, Germany) on random visual fields/slide at a magnification of 15,000× (field area 300 µm^2^).

## 3. Results

### 3.1. Predation Assays on Planktonic Cultures.

Predation assays carried out in LF82 planktonic growth showed a predatory activity of *B. bacteriovorus* against the *E. coli* LF82 strain. 

**Figure 1:**
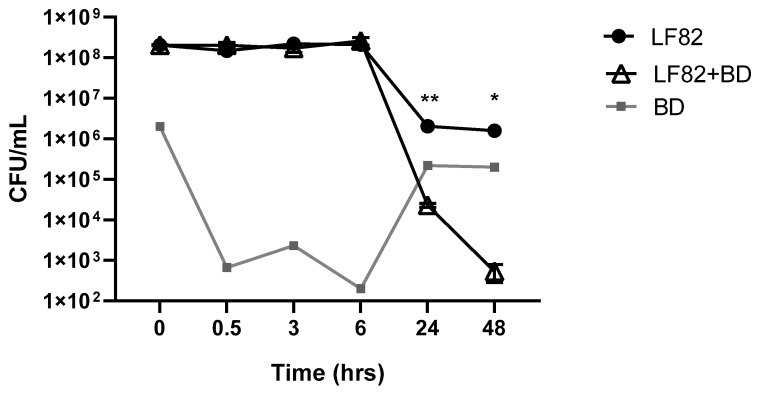
Predation assay of *Bdellovibrio bacteriovorus* on Adherent-Invasive *Escherichia coli* (AIEC) strain LF82 broth culture. The graph displays the CFU/mL of LF82 in co-culture with *B. bacteriovorus* and of the control culture of LF82 at different time points. The PFU/mL of *B. bacteriovorus* at the same time points is also shown. All experiments were conducted three times independently. Statistical analysis performed using two-way ANOVA with Holm–Sidak’s multiple comparison post-test showing a significant reduction of LF82 load at 24 and 48 h (* *p* = 0.0418; ** *p* = 0.0016).

The LF82 viability was evaluated at 30 min, 3 h, 6 h, 24 h, and 48 h of prey and predator co-culture in Ca/HEPES broth and compared to LF82 viability grown alone in the same medium. As shown in Figure 1, a significant decrease of LF82 count was observed in the co-culture with the predator *B. bacteriovorus* at 24 and 48 h (*p* = 0.0016 and *p* = 0.04, respectively), along with a significant increase of *B. bacteriovorus* concentration. Similar results were obtained with the nonpathogenic *E. coli* MG1655, indicating that the predator *B. bacteriovorus* is also able to attack the new pathotype AIEC LF82 strain. The fact that *B. bacteriovorus* is a predator of Gram-negative bacteria, regardless of the strain pathogenicity, is corroborated by this result.

### 3.2. B. bacteriovorus Impact on Preformed LF82 Biofilm and on Biofilm Development

**Figure 2:**
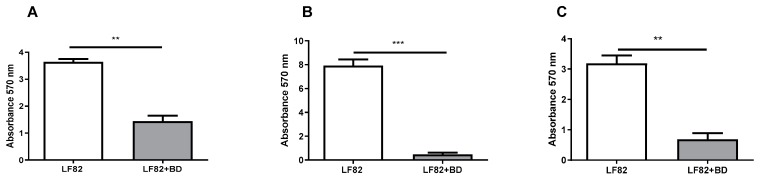
*B. bacteriovorus* predation assay on LF82 biofilm. (**A**) 72-h-old preformed LF82 biofilm was treated with the predator (BD) for 24 h (grey bar); as a control, 72-h-old LF82 biofilm was not exposed to BD (white bar), (*p* = 0.0022). (**B**) biofilm formation by LF82 after 48-h exposure to *B. bacteriovorus* (grey bar); LF82 control untreated biofilm (white bar), (*p* < 0.0001). (**C**) biofilm formation by LF82 after 72-h exposure to *B. bacteriovorus* (grey bar); LF82 control untreated biofilm (white bar), (*p* = 0.0012). All experiments were conducted three times independently. Statistical analysis was performed using Mann–Whitney test. The amount of asterisks (*) obtained from GraphPad Prism software is directly related to statistical significance.

The impact on preformed AIEC biofilm was evaluated by treating a 72-h-old biofilm with a suspension of *B. bacteriovorus* (10^8^ PFU/mL) for 24 h. A significant biofilm biomass reduction (*p* = 0.00220) was observed when compared to untreated LF82 preformed (Figure 2, panel A). *B. bacteriovorus* was able to prevent the development of LF82 biofilm when a co-culture of predator and prey was used to inoculate 96-well plates. LF82 biofilm formation was significantly lower, both at 48 and 72 h, compared to the untreated control (*p* < 0.0001, and *p* = 0.0012 respectively), as can be seen in Figure 2B,C. The strain MG1655 was not a biofilm producer.

### 3.3. B. bacteriovorus Impact on LF82 Adhesion/Invasion Ability on Caco-2 Cell Line

**Figure 3:**
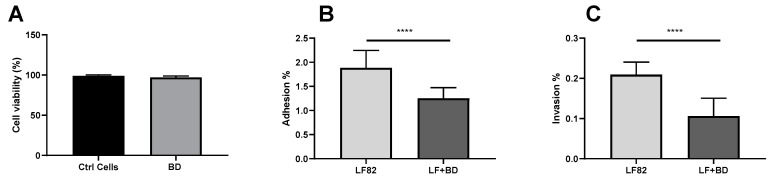
Evaluation of the cytotoxicity of *B. bacteriovorus* on Caco-2 cell line and the effect of *B. bacteriovorus* on LF82 adhesion and invasion into Caco-2 cells. (**A**) Cell viability (%) of Caco-2 cell monolayer not pretreated (black bar) and pretreated with *B. bacteriovorus* (grey bar) after trypan blue dye exclusion assay (Mann–Whitney *p* = 0.0631). (**B**) LF82 adhesion level on Caco-2 cell line pre-treated with *B. bacteriovorus* (dark grey bar); LF82 adhesion level on untreated Caco-2 cell line (grey bar) *(p* < 0.0001, pre-treated vs. untreated). (**C**) LF82 invasion level on Caco-2 cell line pre-treated with *B. bacteriovorus* (dark grey bar); LF82 invasion level on untreated Caco-2 cell line (grey bar) (*p* < 0.0001, pre-treated vs. untreated). All experiments were conducted three times independently. Statistical analysis was performed using Mann–Whitney test. The amount of asterisks (*) obtained from GraphPad Prism software is directly related to statistical significance.

The impact of *B. bacteriovorus* on the adhesiveness and invasiveness of LF82 was assessed on Caco-2 cells pre-treated with a suspension of the predator. A significant decrease of LF82 adhesion and invasion on *B. bacteriovorus* pre-treated Caco-2 cells was observed (*p* < 0.0001 for both) compared with LF82-infected cells that were not pretreated (Figure 3A,B). As reported in [12], in untreated cells infected only with the non-adhesive and noninvasive strain MG1655, we observed that the mean adhesion rate was 0.4% that of the original inoculum.

### 3.4. In Vivo Predatory Activity of B. bacteriovorus on AIEC

**Figure 4:**
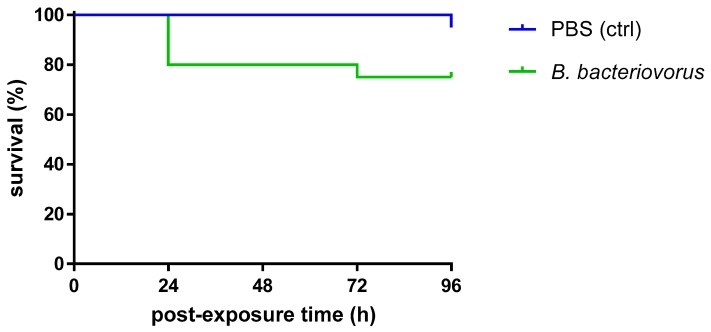
Effect of the injection of *B. bacteriovorus* (1.0 × 10^5^ PFU/larva) on the survival of *Galleria mellonella* larvae. Control larvae were administered with the vehicle (PBS) alone. The larval survival over 96 h was not significantly different (*p* = 0.0738) compared with that observed in control larvae, as assessed by the log-rank (Mantel–Cox) test. Each group (*n* = 20 larvae) was tested two times independently.

**Figure 5:**
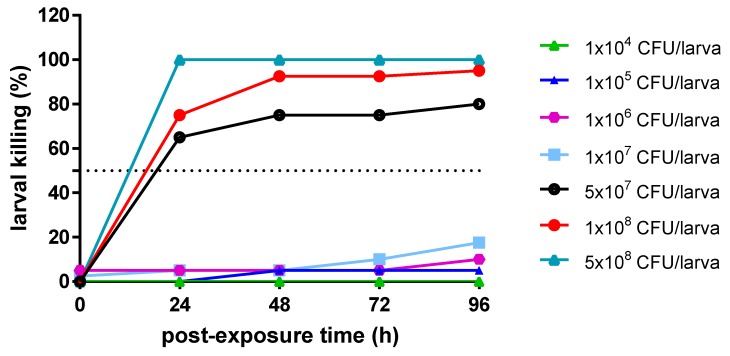
Dose–effect curve. Each larva was administered with *E. coli* LF82 at several doses (10^4^, 10^5^, 10^6^, 10^7^, 5 × 10^7^, 10^8^, and 5 × 10^8^ CFU/larva). Control larvae were administered with the vehicle (PBS) alone. The larva survival was monitored daily over 96 h. Each group (*n* = 20 larvae) was tested two times independently.

**Figure 6:**
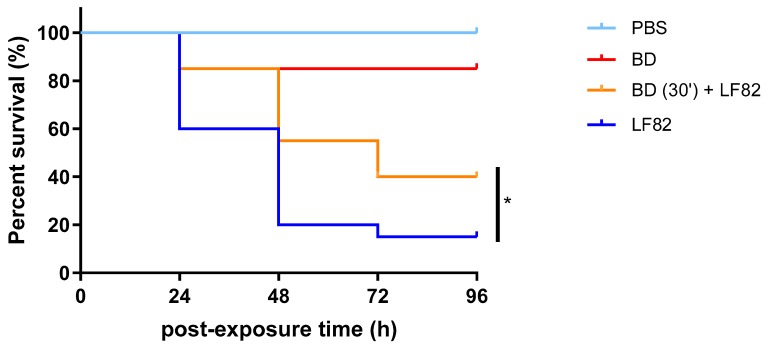
Protective effect of *B. bacteriovorus* against *E. coli* LF82G systemic infection in *G. mellonella* larvae. Thirty minutes after infection of *B. bacteriovorus* at 1.0 × 10^5^ PFU/larva, a lethal dose (LD_50_: 5.0 × 10^7^ CFU/larva) of *E. coli* LF82 was administered to each larva. Positive control larvae were injected with PBS and 30 min later with *E. coli* LF82. Negative control larvae were administered twice with the vehicle (PBS) alone, 30 min apart. The pre-exposure to *B. bacteriovorus* significantly protected *G. mellonella* from the *E. coli* infection, as assessed by the log-rank (Mantel–Cox) test (*p* = 0.0283). Each group (*n* = 20 larvae) was tested two times independently.

*B. bacteriovorus* is not toxic for *G. mellonella*. The positive correlation found between mammalian virulence factors to those isolated in *G. mellonella* wax moth makes this insect a suitable host model for studying microbial pathogenesis. In order to evaluate the toxicity of the predatory bacteria in *G. mellonella*, larvae were injected with *B. bacteriovorus* at 1.0 × 10^5^ PFU/larva, and the larval viability was monitored daily over 96 h (Figure 4). The viability rates of the worms were 80% at 24 and 48 h, and decreased to 75% after 72 h post infection. No deaths were observed in any of the uninfected control larvae over 96 h. The log-rank (Mantel–Cox) test showed that no differences were found among survival rates. Pre-exposure to *B. bacteriovorus* had prophylactic activity against *E. coli* LF82 infection in *G. mellonella*. The dose–effect curve showed that *E. coli* LF82 caused a dose-dependent larval killing; particularly, LD_50_ after 24 h was 5.0 × 10^7^ CFU/larva (Figure 5). The protection associated with a prophylactic *B. bacteriovorus* administration against *E. coli* LF82 infection was then evaluated in a *G. mellonella* model. Preliminary results indicated that pre-exposure to *B. bacteriovorus* significantly prolonged the survival of *G. mellonella* larvae (*p* = 0.0283; Mantel–Cox test), compared with control larvae, by 25% (after 24, 72, and 96 h) and 35% (after 48 h) (Figure 6).

### 3.5. Field Emission Scanning Electron Microscopy

**Figure 7:**
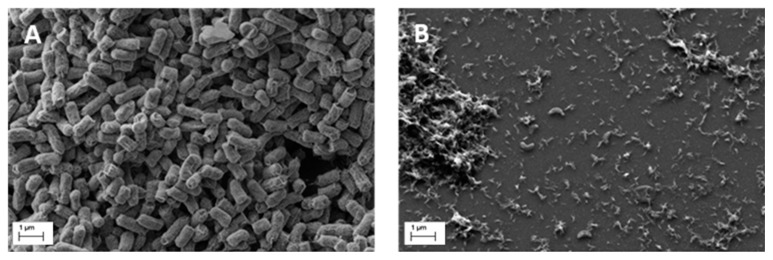
FESEM images. (**A**) 72-h-old LF82 biofilm treated with PBS for 24 h; (**B**) 72-h-old LF82 biofilm treated with *B. bacteriovorus* in PBS for 24 h.

FESEM allowed us to highlight *B. bacteriovorus* predatory activity on preformed LF82 biofilm. A 72-h-old LF82 biofilm, formed on two silicon slides (Figure 7A,B), was treated one with a suspension of *B. bacteriovorus* (10^8^ PFU/mL) in PBS for 24 h (Figure 7B), the other one with PBS for 24 h (Figure 7A). After treatment, a clear and significant reduction of biofilm was observed in the slide treated with *B. bacteriovorus* (Figure 7B). The images indicate a reduction of LF82 biofilm, corroborating the results obtained with the predation assay in sessile growth form (Figure 2).

## 4. Discussion

In their natural environment, bacteria are subjected to predation by bacteriophages and prokaryotic predators [21]. *B. bacteriovorus* is a microorganism naturally present in environmental microbial ecosystems, where it exerts a control on the Gram-negative bacterial populations’ growth rate. Due to the amphibiotic nature of *B. bacteriovorus* and to the difficulties connected to antibiotic resistance, this bacterial predator has mostly been investigated as a possible alternative to antibiotics [26,33]. Shatzkes and collaborators [34] demonstrated *B. bacteriovorus*’ ability to attenuate the bacterial burden of *Klebsiella pneumoniae*, an important human pathogen. The potential of *B. bacteriovorus* has mainly been evaluated as a biological antibacterial agent for periodontal [35], lung [28], and ocular infections [36]. Atterbury et al. demonstrated that orally administered *Bdellovibrio* species in young chickens are able to survive in gut conditions for long enough to have a beneficial therapeutic effect against *Salmonella enteritidis* infection [37]. However, resistance phenomena of the preys against the predator *B. bacteriovorus* should not be underestimated. Several studies showed that changes in the outer-membrane composition and the presence of the bacterial capsule do not completely avoid *Bdellovibrio* predation, but further investigations are required [38,39]. The study of Shemesh and Jurkevitch shows an increased prey resistance to predation after exposure to the predators. However, the resistance seems to be a phenotypic plastic response rather than the result of mutations—it seems to be a transient response of the population due to the presence of the stress (the predator) [40].

The study from Atterbury [37] also showed that, although a temporary increase of Gram-positive bacteria in *Bdellovibrio*-treated birds was detected, *Bdellovibrio* administration did not lead to detrimental effects on the health of noninfected birds. A recent study showed that, in intranasally and intravenously inoculated mice, there was a temporary increase in pro-inflammatory cytokines and chemokines (IL-1β, IL-6, IL-23, CXCL-1/KC, IFN-γ, and TNF-α). However, their levels went back to baseline in 18–24 h, indicating that a strong or persistent immune response is not induced by the predator [34]. Our previous study [22] revealed the presence of *B. bacteriovorus* in the intestinal mucosae of healthy subjects, and its absence in CD patients’ intestinal mucosae. *B. bacteriovorus* in healthy subjects would seem to play the same role it does in natural microbial ecosystems, by controlling Gram-negative bacterial growth. Its absence in CD patients could be one of the causes underlying the observed Gram-negative bacterial overgrowth, like the AIEC strain [41].

To date, several strategies focused on AIEC growth control and reduction on intestinal mucosae are under study, such as the use of antibiotics, the attempt to block AIEC’s adhesion ability by preventing the interaction between FimH and CEACAM6 [18,19], and the attempt to block AIEC’s cell-invasion ability using the Gp96 chaperone or the OmpA trans-membrane proteins as targets [20,42,43,44].

In the present work we have evaluated the potentiality of the predator bacteria *B. bacteriovorus* as therapeutic candidate to counteract the growth of AIEC strains. Our results indicate a predatory activity of *B. bacteriovorus* against AIEC strain LF82, both in sessile and in planktonic growth forms. The ability of *B. bacteriovorus* to destroy bacterial biofilm could be of extraordinary therapeutic value, since most human infections are sustained by biofilm-associated pathogens [45]. We established the predatory activity against the new pathotype of *E. coli*, the AIEC strain. In natural habitats, the predator’s role is to maintain balanced relationships among the different species that are part of complex ecosystems like the gut microbiota. When Gram-negative overgrowth takes place, as in the inflamed intestinal mucosae of CD patients, *B. bacteriovorus* brings the ecosystem back to a balanced state, re-establishing the amount of prey. The prey–predator model of Lotka-Volterra shows how, in a balanced ecosystem, prey and predator populations are characterized by continuous oscillations, with a greater oscillation value for preys. The non-eradication of prey by *Bdellovibrio*-like organisms (BLOs) has been documented in several studies with different preys [40,46], therefore suggesting that killing the entire prey population would be disadvantageous for the predator.

A significantly lower level of LF82 adhesion and invasion in the Caco-2 intestinal cell line pre-treated with suspension of *B. bacteriovorus* was obtained. The decreased adhesiveness and invasiveness detected could be caused by the predatory activity of *B. bacteriovorus*, although further investigations are required to confirm this. As can be seen in Figure 1, no predatory activity was observed after only two hours of co-culture (predator and prey), so the observed lowering of LF82 adhesion-invasion could also be due to a decrease of available epithelial cells adhesion sites to which *B. bacteriovorus* cells could be bound in the pre-treatment phase. Results on the animal model *G. mellonella* seem to suggest that *B. bacteriovorus* is not toxic to the larvae, as observed in vitro in the Caco-2 cell monolayer, confirming previous findings [36]. Furthermore, pre-treatment of *G. mellonella* with the predator *B. bacteriovorus* seemed to have a protective effect against AIEC infection.

Several published studies reported that *G. mellonella*, although it does not replace well-established mammalian models, represents an attractive model organism for the study of microbial pathogenicity, due to its amenability to infection and its ability to mount an innate immune response. In fact, several studies have focused on various pathogenic bacteria, such as *Burkholderia cepacia* [47], *Acinetobacter baumannii* [48], *Pseudomonas aeruginosa* [49], and *Enterococcus faecalis* [50]. Although no data were published in this regard with adherent-invasive (AIEC) strains, recent works indicated that *G. mellonella* is a valuable model to study enteropathogenic (EPEC) and enteroaggregative (EAEC) *E. coli* virulence [51,52,53]. Several analogies were in fact observed between the epithelial cells of insect larval midguts and intestinal cells of mammalian digestive systems [54]. Besides, the survival of *G. mellonella* at 37 °C allows the investigation of temperature-dependent virulence factors, and its immune system shares a high degree of structural and functional similarities to the innate immune system of vertebrates, since hemocytes are analogous to human phagocytes [52]. In addition to the hemocyte-mediated cellular immune response, *G. mellonella* also has a humoral immune response. The current limitation of the *G. mellonella* model is the lack of both universal larval genotypes and the standardization for the conditions used for feeding, reproduction, and maintenance of the animals that might lead to quantitative differences in the results.

Finally, results from FESEM analysis substantiate the predatory activity of *B. bacteriovorus* towards preformed AIEC biofilm. Since the two FESEM slides underwent exactly the same preparatory procedure, including the treatment with PBS, even if a purely accidental detachment of the biofilm cannot be excluded, we believe that the observed biofilm reduction reported in Figure 7B is mainly due to the predatory activity of *B. bacteriovorus*.

A feature that is constantly present in intestinal dysbiosis is the loss of microbiota diversity (LOMD) [55]. In CD patients, a dysbiosis status and a LOMD is reported [56,57]. The LOMD in CD could be the consequence of the reduction/loss of bacterial predation. When bacterial predators like *B. bacteriovorus* are missing, some species (e.g., “pathobiont” bacteria, like AIEC strains) can speedily grow, replacing other species such as *Faecalibacterium prausnitzii* within the Firmicutes phylum and accessible resources would be limited [58].

Overall, our results clearly indicate that *B. bacteriovorus* could be a good candidate to control AIEC strains at the mucosal level and, consequently, to restore a “healthy” gut microbial ecosystem in CD patients.
